# Hybrid stochastic framework predicts efficacy of prophylaxis against HIV: An example with different dolutegravir prophylaxis schemes

**DOI:** 10.1371/journal.pcbi.1006155

**Published:** 2018-06-14

**Authors:** Sulav Duwal, Laura Dickinson, Saye Khoo, Max von Kleist

**Affiliations:** 1 Department of Mathematics & Computer Science, Freie Universität Berlin, Berlin, Germany; 2 Institute of Translational Medicine, University of Liverpool, Liverpool, United Kingdom; Duke University, UNITED STATES

## Abstract

To achieve the 90-90-90 goals set by UNAIDS, the number of new HIV infections needs to decrease to approximately 500,000 by 2020. One of the ‘five pillars’ to achieve this goal is pre-exposure prophylaxis (PrEP). Truvada (emtricitabine-tenofovir) is currently the only medication approved for PrEP. Despite its advantages, Truvada is costly and requires individuals to adhere to the once-daily regimen. To improve PrEP, many next-generation regimen, including long-acting formulations, are currently investigated. However, pre-clinical testing may not guide candidate selection, since it often fails to translate into clinical efficacy. On the other hand, quantifying prophylactic efficacy in the clinic is ethically problematic and requires to conduct long (years) and large (N>1000 individuals) trials, precluding systematic evaluation of candidates and deployment strategies. To prioritize- and help design PrEP regimen, tools are urgently needed that integrate pharmacological-, viral- and host factors determining prophylactic efficacy. Integrating the aforementioned factors, we developed an efficient and exact stochastic simulation approach to predict prophylactic efficacy, as an example for dolutegravir (DTG). Combining the population pharmacokinetics of DTG with the stochastic framework, we predicted that plasma concentrations of 145.18 and 722.23nM prevent 50- and 90% sexual transmissions respectively. We then predicted the reduction in HIV infection when DTG was used in PrEP, PrEP ‘on demand’ and post-exposure prophylaxis (PEP) before/after virus exposure. Once daily PrEP with 50mg oral DTG prevented 99–100% infections, and 85% of infections when 50% of dosing events were missed. PrEP ‘on demand’ prevented 79–84% infections and PEP >80% when initiated within 6 hours after virus exposure and continued for as long as possible. While the simulation framework can easily be adapted to other PrEP candidates, our simulations indicated that oral 50mg DTG is non-inferior to Truvada. Moreover, the predicted 90% preventive concentrations can guide release kinetics of currently developed DTG nano-formulations.

## Author summary

In 2012, pre-exposure prophylaxis (PrEP) with Truvada was approved. It is considered one of the ‘five pillars’ by UNAIDS to drastically reduce HIV transmission. However, Truvada provides imperfect protection, is costly and individuals often fail to adhere to the once-daily regimen. Next-generation PrEP compounds, including long-acting formulations, are currently developed to improve PrEP. However, clinical trials using next-PrEP often fail. Since they involve many (>1000) individuals and long durations this incurs unacceptable costs, apart from individual tragedies. While animal- and *ex vivo*/*in vitro* experiments poorly translate into human efficacy, predictive tools are urgently needed that allow for PrEP candidate prioritisation. We developed an efficient simulation tool to predict the prophylactic utility of arbitrary dosing regimen. After developing population pharmacokinetic models for dolutegravir (DTG), we set out to predict its prophylactic utility in PrEP, PrEP ‘on demand’ and post-exposure prophylaxis. We found that 50mg DTG is non-inferior to Truvada in all aforementioned prophylaxis schemes. Moreover, we determined concentrations-prophylaxis profiles, which can guide release kinetics of currently developed DTG nano-formulations.

## Introduction

HIV-1 continues to be one of the greatest public health challenges. While it is possible to suppress virus replication with antiretroviral combination treatment, the virus can persist in cellular and anatomical reservoirs for decades, precluding a cure [1–3]. Because of the inability to cure HIV, preventing its transmission is of utmost importance. The 90-90-90 target formulated by UNAIDS aim to end AIDS by 2030. An intermittent goal will be a drastic reduction of new HIV infections: While approximately 2.1 million individuals became infected with HIV in 2015 [4], the intention is to reduce this number to 500,000 cases by 2020 and to fewer than 200,000 by 2030.

Currently, pre-exposure prophylaxis (PrEP) for high-risk individuals is one of the five ‘pillars’ set by UNAIDS to achieve a drastic reduction in HIV infections. Of the available agents, tenofovir and emtricitabine (Truvada) have been extensively studied and were approved by the FDA and EMEA in 2012 and 2016 respectively. Most studies agree that Truvada can potently prevent HIV infection, if individuals adhere to the once-daily regimen [5, 6]. However, major shortcomings of Truvada-based PrEP are its costs [7], the fact that it imperfectly protects from infection, and the necessity for daily drug intake, which often leads to inadequate adherence. These deficits of Truvada-based PrEP may be overcome by next-generation PrEP regimen, including more cost-efficient drugs, and drug formulations that require antiviral injections only every few month (see [8] for an overview of the PrEP pipeline).

Quantifying prophylactic efficacy in the clinic is ethically problematic and extremely expensive, since it requires to conduct large (N > 1000 individuals) trials over very long time spans (years) to obtain statistically evaluable results. On the other hand, *pre-clinical* PrEP experiments only allow to study certain aspects in isolation. While PrEP efficacy is the result of a multivariate interplay of viral- and host factors there is a lack of translational tools to integrate available knowledge and to rationalize which agents and dosing regimen to test clinically. Having predictive models at hand that allow to rule out-, or prioritize certain regimen can avoid putting individuals at harm and greatly reduce clinical failure rates and associated costs.

Our intention was to develop a method that integrates pharmacokinetic and pharmacodynamic (PK/PD), as well as viral characteristics to *a priori* assess the *per contact* prophylactic efficacy of arbitrary PrEP strategies against HIV.

Recently, modelling approaches have been developed to predict the per-contact PrEP efficacy [9, 10] by integrating various host and viral factors. Despite their advantages, these approaches conventionally neglect the pharmacokinetic–pharmacodynamic characteristics of HIV drugs and are therefore unable to simulate drug dosing, and dose frequency in order to ascertain how PrEP can most effectively be deployed. We recently developed a novel approach which fully integrates the pharmacology of nucleotide reverse transcriptase inhibitors (NRTIs) [11]. This approach, however, approximates virus extinction by its elimination probability during the first replication cycle. While this assumption is reasonable for moderately potent prophylactic compounds (like all investigated NRTIs), it underestimates the prophylactic efficacy of highly potent drugs and fails to predict efficacy in post-exposure prophylaxis (PEP). We overcome the aforementioned limitations by building on recent developments for the simulation of stochastic processes [12], implementing a numerically exact simulation approach to assess PrEP/PEP efficacy for time-varying drug concentrations (pharmacokinetics). The benefit of such integrative framework is to elucidate the relative importance of the distinct pharmacological and viral factors, such as the on- and offset of prophylactic protection, sensitivity to missed dosing events, -virus inoculum size and -timing of virus exposure. In the current work, we combine population pharmacokinetics with the novel stochastic simulation method to analyze different PrEP/PEP schedules with the second-generation integrase inhibitor dolutegravir.

## Methods

The initial replication events after exposure to HIV are highly stochastic. Typically, a low number of founder viruses is responsible for establishing infection [13–16] and the transmission probability per *sexual* exposure [17, 18] is very low. While two types of stochastic noise are typically considered in biology, roughly categorized as internal- vs. external noise [19, 20], we herein focused on the former. This assumes that the stochastic outcome of viral exposure (infection/non-infection) can be explained by the order in which reaction occur. For example, when a single virus comes into proximity of target cells, it may either be cleared or it may infect the target cell which can trigger a systemic infection. Prophylactic drugs shift the balance between these two events in favor of virus clearance. Stochastic dynamics of this type are defined by a multivariate Poisson process. The evolution of the state probabilities given the initial state *x*_0_ is then described by the chemical master equation (CME). For each possible state *x*_*i*_ we have

$$\begin{array}{ccc} \frac{d}{dt}P(X_{t}=x_{i}|X_{0}=x_{0})\; & = & \sum_{k=1}^{K}a_{k}\left(x_{i}-\nu_{k}\right)\cdot P\left(X_{t}=x_{i}-\nu_{k}|X_{0}=x_{0}\right)\\ & & -a_{k}\left(x_{i}\right)\cdot P\left(X_{t}=x_{i}|X_{0}=x_{0}\right), \end{array}$$

for time *t* ≥ 0, with $X_{t}\in {\mathbb{N}}^{s}$ denoting the state of the system (the combination of the number of viruses, infected cells and drug particles), where *s* denotes the overall number of variables (‘species’). In the equation above, the index *k* runs over all reactions and *a*_*k*_, *ν*_*k*_ denote the reaction propensity of the k^th^ reaction and its stoichiometric change vector respectively. A practical problem with the CME is that even for moderately sized systems (*s* small) the *curse of dimensionality* is encountered and consequently the CME is intractable [21].

### Hybrid formulation

It has been shown that intrinsic stochastic fluctuations may be negligible in the so-called large copy number regimen (when *X*_*t*_ >> 1) and consequently, an ODE approach that models concentrations of molecules *X*_*t*_/Ω, suffices [22]. In the current work, we re-formulate the above stated CME into a hybrid stochastic-deterministic (discrete-continuous) system *X*_*t*_ = (*Y*_*t*_; *Z*_*t*_), where *Y*_*t*_ denotes the discrete-stochastic and *Z*_*t*_ denotes the continuous-deterministic part. In our application, we view the antiviral pharmacokinetics (concentration time profile) as the external dynamical environment *Z*_*t*_, which we will model in terms of a low-dimensional set of ODEs (exemplified below). This is common practice in the pharmacometrics/systems pharmacology field and is supported by the fact that typically large quantities of drug molecules reach the target site. The target site concentrations *D*_*t*_ ⊆ *Z*_*t*_ affect reaction propensities of our internal stochastic system, which models the stochastic events after exposure (viral replication and clearance reactions). The internal system *Y*_*t*_ represents the state of the viral compartments represented by the number of free viruses *V*, early stage infected T cells *T*_1_ and late infected T cells *T*_2_, i.e *Y*_*t*_ = [*V*, T_1_, T_2_,]^*T*^, exemplified below.

While this reduces the dimensionality, propensity functions of the stochastic sub-system are subsequently time-dependent, i.e. the continuous-deterministic drug concentration-time profile may affect reaction rates of the discrete-stochastic subsystem *Y*_*t*_ on an infinitesimally small time scale. We will tackle this problem algorithmically, enabling the numerically exact estimation of prophylactic efficacy.

### Prophylactic efficacy of a drug regimen

Our goal is to estimate the prophylactic efficacy *φ* of particular medication regimen *S*_*D*_. The prophylactic efficacy denotes the reduction in infection probability *per contact*,

$$\begin{array}{c} \varphi \left(Y_{0},S_{D}\right)=1-\frac{1-P_{E}\left(Y_{0}|S_{D}\right)}{1-P_{E}\left(Y_{0}|⌀\right)}\;\;\left(\text{prophylactic}\;\text{efficacy}\right), \end{array}$$ (1)

where *P*_E_(*Y*_0_|*S*_*D*_) and *P*_E_(*Y*_0_|⌀) denote the virus extinction probabilities for a particular prophylactic regimen *S*_*D*_ and in the absence of prophylactic drugs ⌀ respectively. The extinction probability is defined as

$$\begin{array}{c} P_{E}\left(Y_{0}\right)≔P(Y_{t}=[\begin{array}{c} 0\\ 0\\ 0 \end{array}]\;|\;Y_{0}=[\begin{array}{c} V\\ T_{1}\\ T_{2} \end{array}]) \end{array}$$ (2)

for *t* → ∞ and where *Y*_0_ denotes the initial state of the stochastic viral dynamics subsystem. In other words, the extinction probability is the probability that a stochastic trajectory eventually reaches the absorbing state [0, 0, 0]^*T*^ of the viral subsystem *Y*. Naturally, the infection probability is the complement of the extinction probability, *P*_I_(*Y*_0_) = 1 − *P*_E_(*Y*_0_). The terms *P*_E_(*Y*_0_|*S*_*D*_), *P*_E_(*Y*_0_|⌀) will be computed using a mathematical model of the viral dynamics that mechanistically considers the direct effects of antivirals on their respective target processes (outlined in a related article [23]), as well as individual drug pharmacokinetics following particular prophylaxis regimen.

### Viral dynamics (stochastic part)

We adopted the viral dynamics model described in [24, 25]. Long-lived and latently infected cells are only implicitly considered (outlined at the end of the section), motivated by the observation that transmitted viruses are not macrophage-tropic [26, 27] and in line with related modelling approaches [9, 10, 28–30]. Although this model is a coarse representation of the molecular events happening during virus replication, it allows to accurately and mechanistically describe the effect of all existing antiretroviral drug classes on viral replication, as demonstrated in e.g. [31], and can be parameterized by *in vitro* and *clinical* data, Table 1. The modelled viral replication cycle consists of free infectious viruses *V*, uninfected T-cells, early infected T-cells (T_1_) and productively infected T-cells (T_2_). Early infected T-cells (T_1_) and productively infected T-cells (T_2_) denote T-cells prior- and after proviral integration respectively, where the latter produces virus progeny. During the onset of infection the number of viruses is relatively low and the number of uninfected T-cells T_u_ is fairly unaffected by virus dynamics [28, 32, 33]. We thus consider T_u_ = λ_T_/*δ*_T_ to be constant over the course of simulations. The dynamics of the stochastic viral replication model after virus exposure are then defined by six reactions (the model is depicted in S1 Fig):

$$\begin{array}{ccc} a_{1}= & (\text{CL}+{\text{CL}}_{T}\cdot T_{u})\cdot V_{t} & \left(\text{clearance}\;\text{of}\;\text{free}\;\text{virus;}\;V\to *\right) \end{array}$$ (3)

$$\begin{array}{ccc} a_{2}= & (\delta_{\text{PIC}}+\delta_{T_{1}})\cdot {T_{1}}_{,t} & \left(\text{clearance}\;\text{of}\;\text{early}\;\text{infected}\;\text{cell;}\;T_{1}\to *\right) \end{array}$$ (4)

$$\begin{array}{ccc} a_{3}= & \delta_{T_{2}}\cdot {T_{2}}_{,t} & \left(\text{clearance}\;\text{of}\;\text{late}\;\text{infected}\;\text{cell;}\;T_{2}\to *\right) \end{array}$$ (5)

$$\begin{array}{ccc} a_{4}= & \beta \cdot T_{u}\cdot V_{t} & \left(\text{infection}\;\text{of}\;\text{a}\;\text{susceptible}\;\text{cell;}\;V\to T_{1}\right) \end{array}$$ (6)

$$\begin{array}{ccc} a_{5}\left(D_{t}\right)= & \left(1-\eta_{D}\left(t\right)\right)\cdot k\cdot {T_{1}}_{,t} & \left(\text{proviral}\;\text{integration;}\;T_{1}\to T_{2}\right) \end{array}$$ (7)

$$\begin{array}{ccc} a_{6}= & N_{T}\cdot {T_{2}}_{,t} & (\text{production}\;\text{of}\;\text{virus;}\;T_{2}\to V+T_{2}), \end{array}$$ (8)

with ${CL}_{T}=\left(\frac{1}{\rho_{rev}}-1\right)\cdot \beta$ in eq (3), as outlined in [24] where *ρ*_*rev*_ = 0.5 denotes the probability to successfully complete reverse transcription in the absence of inhibitors [34, 35]. Free viruses are cleared by the immune system with a rate constant CL. Further, free viruses can be also cleared during unsuccessful T-cell infection CL_T_ through the destruction of essential viral components of the reverse transcription-, or pre-integration complex [34, 35]. The term *β* represents the lumped rate of infection of T-cells, including the processes of virus attachment to the cell, fusion and reverse transcription, leading to an early infected cell T_1_, before proviral integration. The term *k* denotes the rate by which early infected T_1_ cells are transformed into productively infected T_2_ cells, involving proviral integration and cellular reprogramming. The term N_T_ denotes the rate of production of infectious virus progeny by productively infected T_2_ cells. The terms $\delta_{T_{1}}<\delta_{T_{2}}$ denote the rates of clearance of T_1_ and T_2_ cells respectively and *δ*_PIC_ denotes the rate of intracellular destruction of the pre-integration complex. Parameters for the viral model are summarized in Table 1 and a mechanistic derivation of the dynamics from first principles is given in [24] (Supplementary Text therein). In this article, we study distinct prophylactic schemes with the second-generation integrase inhibitor dolutegravir (DTG). Integrase inhibitors act intracellularly by preventing proviral integration. In our virus dynamics model (eqs (3)–(8)), this translates into a decrease in propensity function *a*_5_ by a factor (1 − *η*_*D*_). Notably, DTG is active in its adminstered form (does not require biotransformation) and has physicochemical attributes that allow the unbound drug to rapidly cross the cellular membrane. We modelled the *direct* effect of dolutegravir using the Emax-equation [36]

$$\begin{array}{c} \eta_{D}\left(t\right)=\frac{D_{t}^{m}}{{\text{IC}}_{50}^{m}+D_{t}^{m}}, \end{array}$$ (9)

where *D*_*t*_ is the target site concentration of the drug at time *t* and the term IC_50_ and *m* denote the drug concentration at which the targeted process is inhibited by 50% and a hill coefficient [37] respectively. Note that the equation above couples the stochastic viral dynamics subsystem *Y*_*t*_ to the deterministic subsystem *Z*_*t*_, where the latter propagates the drug concentrations *D*_*t*_ ⊆ *Z*_*t*_.

**Table 1:** Parameters used for the viral dynamics model. Excerpt from [24], except for CL(naive), which assumed that virus clearance is smaller in virus-naive individuals compared to infected individuals, in line with [55, 87]. All parameters refer to the absence of drug treatment ⌀. All parameters in units [1/day]).

Parameter	Value	Reference
λ_T_	2⋅10^9^	[82]
$\delta_{T},\delta_{T_{1}}$	0.02	[83]
$\delta_{T_{2}}$	1	[84]
*δ*_PIC_	0.35	[35, 85]
*k*	0.35	[35]
*β*	8⋅10^−12^	[86]
N_T_	670	[24, 83]
CL(naive)	2.3	[10, 28]

#### Pharmacodynamic parameters

The hill coefficient *m* and 50% inhibitory concentration IC_50_ have been measured *ex vivo* using single-round infection assays in primary human peripheral blood mononuclear cells, supplemented with 50% human serum [38]. However, the measured IC_50_ has to be corrected for protein binding, since dolutegravir is highly protein bound in human plasma (98.9%) which will be underestimated by the assay (which utilizes 50% human serum). This correction is in line with the widely accepted ‘free drug hypothesis’ [39] that states that the available concentrations at the target site (intracellular space) correspond to the *unbound* moieties [40, 41]. Dolutegravir obeys physico-chemical characteristics to enable the *unbound* drug to rapidly cross cellular membranes, generating an equilibrium between the *unbound* drug on either side of the cellular membrane [42]. However, since the *unbound* fraction *f*_*u*,assay_ in the assay is different to the physiological *unbound* fraction *f*_*u*,plasma_, the measured IC_50_ value needs to be adjusted/scaled. After protein adjustment, we obtain IC_50_ = 89(*CV* = 25.3%) [nM] and *m* = 1.3(*CV* = 15.3%), see related article [23].

### Dolutegravir pharmacokinetics (deterministic part)

We used non-linear mixed effects modelling techniques [43] to derive a *descriptive* pharmacokinetic model that accurately captures the observed pharmacokinetic variability within- and across different patients. In this framework, both a minimal structural model *f*(*θ*_*i*_, ⋅) is fitted to clinical data, alongside with statistical models describing the distribution of pharmacokinetic parameters *θ* within the population, as well as the measurement- or unexplained noise.

Let $D_{i,t}$ be the measured plasma concentration of a drug in the *i*^th^ individual at time point *t*. The likelihood of that measurement is defined through

$$\begin{array}{c} D_{i,t}=f\left(\theta_{i},t\right)\cdot \left(1+\epsilon_{i,t}\right)+{\tilde{\epsilon }}_{i,t} \end{array}$$ (10)

where *f* denotes the solution of the structural model (a low dimensional set of ordinary differential equations; see below) that corresponds to the measurement. The vector *θ*_*i*_ contains the pharmacokinetic parameters for the *i*^th^ individual. The variables *ϵ*_*i*, *t*_ and ${\tilde{\epsilon }}_{i,t}$ denote proportional and additive error terms (measurement- or unexplained noise), which are typically assumed to be normal distributed, i.e. $\epsilon_{i,t}∼N(0,\sigma^{2})$ and ${\tilde{\epsilon }}_{i,t}∼N(0,{\tilde{\sigma }}^{2})$. The *prior* probability is typically assumed to be a multivariate log-normal distribution with

$$\begin{array}{c} \text{log}\;\theta_{i}=\text{log}\;\theta +r_{i}, \end{array}$$ (11)

where *θ* denotes the vector of mean population parameters (fixed effects) and *r*_*i*_ is normal distributed, i.e. $r_{i}∼N(0,\Psi )$.

#### Parameter and model inference

We used dolutegravir concentration-time data from two clinical studies. One study assessed 50mg once daily dolutegravir administered to 17 healthy volunteers for 10 days and serial blood sampling performed up to 216 hours after the final dose [44] (n = 12 female, n = 8 Caucasian). The second study was performed in 39 HIV-infected patients (n = 2 female, n = 27 Caucasian) stable on efavirenz-based therapy (viral load <40 copies/mL), switched to dolutegravir (50mg once daily). Random, single blood samples were drawn over the 24 hour dosing interval 1, 2, 3 and 4 weeks post-switch [45]. Median (range) age, weight and BMI of all individuals were 47 years (26-68), 76 kg (51-105) and 26 kg/m^2^. All data were modelled simultaneously and the first-order estimation (FOCE-I) method of NONMEM (v.7.3, ICON plc, Dublin, Ireland), interfaced with Pirana (v.2.9.0; www.pirana-software.com) was used for parameter inference. One- and two compartment models were explored with differences between hierarchical models assessed by statistical and graphical methods. The minimal objective function value (OFV; equal to -2 log likelihood) was used as a goodness-of-fit diagnostic with a decrease of at least 3.84 units corresponding to a statistically significant difference between nested models (p = 0.05, *χ*^2^ distribution, 1 degree of freedom). Standard errors of the estimates were determined with the COVARIANCE option of NONMEM and individual Bayesian parameter and concentration estimates by the POSTHOC option. Random effects (inter-individual, inter-occasion variability) in model parameters were included if model fit was improved (i.e. ΔOFV ≥-3.84 points). To describe residual variability, proportional, additive and a combined proportional-additive error models were evaluated and the best fitting were carried forward. The effect of residual efavirenz concentrations on dolutegravir clearance was determined by estimating 5 separate fixed effects (CL/F values) for dolutegravir alone in healthy volunteers (study 1) and for weeks, 1, 2, 3, 4, post-switch from efavirenz in HIV-infected patients (study 2). Other covariates assessed in the model included weight, age, body mass index (BMI), sex, ethnicity, HIV status, and food consumption within 3 hours of drug intake. A forwards inclusion-backwards elimination method [46] was used to determine whether there were any important associations between parameter estimates and covariates. Each covariate was introduced separately and only retained in the model if inclusion produced a statistically significant decrease in OFV of at least 3.84 units (*p* ≤ 0.05, *χ*^2^ distribution, 1 degree of freedom) and was biologically plausible. A backwards elimination step was carried out once all relevant covariates were incorporated and covariates retained if removal from the model produced a significant increase in OFV (> 10.83 points; *p* ≤ 0.001, *χ*^2^ distribution, 1 degree of freedom).

#### Final PK model

The final model was a two-compartment model with oral absorption:

$$\begin{array}{ccc} \frac{d}{dt}Z_{1} & = & -k_{a}\cdot Z_{1} \end{array}$$ (12)

$$\begin{array}{ccc} \frac{d}{dt}D=\frac{d}{dt}Z_{2} & = & \frac{k_{a}\cdot Z_{1}}{V_{c}/F_{\text{bio}}}-\frac{\text{CL}/F_{\text{bio}}}{V_{c}/F_{\text{bio}}}\cdot Z_{2}-\frac{Q/F_{\text{bio}}}{V_{c}/F_{\text{bio}}}\cdot Z_{2}+\frac{Q/F_{\text{bio}}}{V_{p}/F_{\text{bio}}}\cdot Z_{3} \end{array}$$ (13)

$$\begin{array}{ccc} \frac{d}{dt}Z_{3} & = & \frac{Q/F_{\text{bio}}}{V_{c}/F_{\text{bio}}}\cdot Z_{2}-\frac{Q/F_{\text{bio}}}{V_{p}/F_{\text{bio}}}\cdot Z_{3}, \end{array}$$ (14)

whereby *Z*_1_ and *Z*_3_ denote the amount of drug in the dosing compartment and the concentration of dolutegravir in the peripheral compartment respectively. The variable of interest is the concentration in the blood plasma (central compartment), i.e. *D* = *Z*_2_. Dosing events were modelled as impulse inputs, with

$$\begin{array}{c} Z_{1,t}=Z_{1,t}+{\text{dose}}_{k}, \end{array}$$ (15)

whenever the current simulation time *t* coincided with a dosing event *τ*_*k*_. In the equations above, *k*_*a*_ and CL/*F*_bio_ denote the uptake and bioavailability-adjusted drug clearance respectively. The term *V*_*c*_/*F*_bio_ and *V*_*p*_/*F*_bio_ are the bioavailability-adjusted volume of the central and peripheral compartment. The term *Q*/*F*_bio_ is the intercompartmental clearance rate adjusted for bioavailability.

### Numerical simulation of hybrid model

As mentioned before, direct computation of the extinction probabilities in eq (1) may not be possible as *Y*_*t*_ still contains a prohibitively large number of states. In the following, we will utilize the results from a related article [23] in combination with the novel EXTRANDE algorithm [12] to compute the extinction probabilities for *time-varying* drug effects, i.e. taking drug pharmacokinetics into account.

We consider *a*_0_(*Y*_*t*_, *D*_*t*_) = ∑_*k*_ *a*_*k*_(*Y*_*t*_, *D*_*t*_) to be the sum of the *K* reaction propensities changing the internal stochastic system. Note that in the exact SSA [47], no external input exists and therefore the propensities stay constant in between two reaction firings. In this case, the time to the next reaction event is exponentially distributed with parameter *a*_0_. In our case *a*_0_(*t*) changes between two stochastic reaction firings because of pharmacokinetic inputs. Solving this problem requires to compute *a*_0_(*t*) by numerical integration each time after a stochastic reaction has fired, which can be computationally expensive. Instead, in EXTRANDE, an upper bound *B* for *a*_0_(*t*) is estimated and thinning techniques (rejection steps) are employed. For a look-ahead time horizon *L*, the upper bound *B*_*t*+*L*_ is chosen, such that

$$\begin{array}{c} B_{t+L}\geq a_{0}(Y_{t+u},D_{t+u}) \end{array}$$ (16)

holds for all *u* ≤ *L* and assuming no stochastic reaction fires within the time interval *t* + *L*. When *L* is fixed, it is possible to solve for *D*_*t*+*L*_, since it is assumed that stochastic reactions do not affect *D*. E.g. if the dynamics of *D* ⊆ *Z* are determined by a set of ordinary differential equations, numerical integration from *t* to *t* + *L* enables to predict *D*_*t*+*L*_, which in turn allows to compute *a*_0_(*Y*_*t*+*u*_, *D*_*t*+*u*_).

The internal stochastic system is then augmented with an extra reaction (a *K*+1th reaction), whose firing does not change the state of the stochastic subsystem *Y*. The probability of firing this extra reaction at time *t* + *L* is proportional to the ratio $\frac{B_{t+L}-a_{0}(Y_{t+u},D_{t+u})}{B_{t+L}}$.

Obviously, it has to be guaranteed that eq (16) is true for the entire look-ahead time horizon *L*. On the other hand when *B*_*t*+*L*_ is chosen to be too large, many extra reactions will be fired (rejection/thinning step) and the algorithm becomes inefficient. A key to efficient simulation with EXTRANDE is therefore a good choice of *B*_*t*+*L*_, which in turn depends on the look-ahead time horizon *L*.

#### Upper bound *B* and look-ahead horizon *L*

From equation eq (9) it is clear that (1 − *η*_*D*_) ∈ [0, 1] and consequently

$$\begin{array}{c} a_{0}\left(Y_{t+u},D_{t+u}\right)\leq a_{0}\left(Y_{t},⌀\right), \end{array}$$ (17)

for any time interval 0 ≤ *u* ≤ *τ* *before* a stochastic reaction has fired where parameter ⌀ denotes the absence of drugs. Consequently, we used *B* = *a*_0_(*Y*_*t*_, ⌀) throughout the article as an upper bound to meet condition *a*_0_(*Y*_*t*+*u*_, *D*_*t*+*u*_) ≤ *B* without the requirement to select a look-ahead time horizon *L*.

#### Classification of trajectories

When using EXTRANDE to assess the PrEP/PEP efficacy, we are particularly interested in classifying stochastic trajectories as *extinction* or *infection* events. The virus dynamics model has an absorbing state *Y*_*t*_ = [0, 0, 0]^*T*^ corresponding to virus extinction. Whenever trajectories hit this state we can stop the simulation. To stop the simulation when trajectories move away from the extinction state is not a straightforward choice.

Given a user-defined threshold *ε* << 1 we only consider stochastic states within an *extinction simplex*, e.g. states *Y* for which

$$\begin{array}{c} P_{E}\left(Y_{t},D_{\text{max}}\left(t\right)\right)\geq \varepsilon \end{array}$$ (18)

is true, i.e. extinction can occur with a probability greater *ε*. Simulations are consequently stopped whenever *P*_E_(*Y*_*t*_, *D*_max_(*t*)) < *ε*, where *D*_max_(*t*) = max_*u*∈[*t*, ∞]_ *D*_*u*_ denotes the maximum achievable drug concentration in [*t*, ∞] to be pre-computed from a pharmacokinetic trajectory. This criterium guarantees that the numerical error in classifying trajectories as infection events stays below the user defined criteria *ε* << 1.

In a related article [23], we derived analytical solutions for computing the extinction probability for any particular state of the virus dynamics system, under the assumption that the drug concentrations *D* were constant (for computing the *extinction simplex*, we use *D* = *D*_max_(*t*)):

$$\begin{array}{ccc} {\text{log}}_{10}(P_{E}\left(Y,D\right)) & = & V\cdot {\text{log}}_{10}(P_{E}\left(\hat{V},D\right))+T_{1}\cdot {\text{log}}_{10}(P_{E}\left({\hat{T}}_{1},D\right))+T_{2}\cdot {\text{log}}_{10}(P_{E}\left({\hat{T}}_{2},D\right)). \end{array}$$

where $P_{E}(Y_{0}=\hat{V})$, $P_{E}(Y_{0}={\hat{T}}_{1})$ and $P_{E}(Y_{0}={\hat{T}}_{2})$ denote the extinction probabilities when only one virus, one early- or one productively infected cell was present, and V, T_1_ and T_2_ denote the number of viruses, early- and late infected cells. These terms can be further decomposed (see related article [23]) into

$$\begin{array}{ccc} P_{E}\left(Y_{0}=\hat{V}\right) & = & \min(1,1-\frac{a_{4}}{a_{1}+a_{4}}\cdot \frac{a_{5}\left(D\right)}{a_{2}+a_{5}\left(D\right)}\cdot (1-\frac{1}{R_{0}\left(V,D\right)})) \end{array}$$ (19)

$$\begin{array}{ccc} P_{E}\left(Y_{0}={\hat{T}}_{1}\right) & = & \min(1,1-\frac{a_{5}\left(D\right)}{a_{2}+a_{5}\left(D\right)}\cdot (1-\frac{1}{R_{0}\left(V,D\right)})) \end{array}$$ (20)

$$\begin{array}{ccc} P_{E}\left(Y_{0}={\hat{T}}_{2}\right) & = & \min(1,\frac{1}{R_{0}\left(V,D\right)}), \end{array}$$ (21)

where $R_{0}(V,D)=\frac{a_{4}}{a_{1}+a_{4}}\cdot \frac{a_{5}(D)}{a_{2}+a_{5}(D)}\cdot \frac{a_{6}}{a_{3}}$ is the reproductive number in the presence of drug *D*, i.e. the expected number of viruses emerging from a single parent virus in one replication cycle.

**Fig 1:**
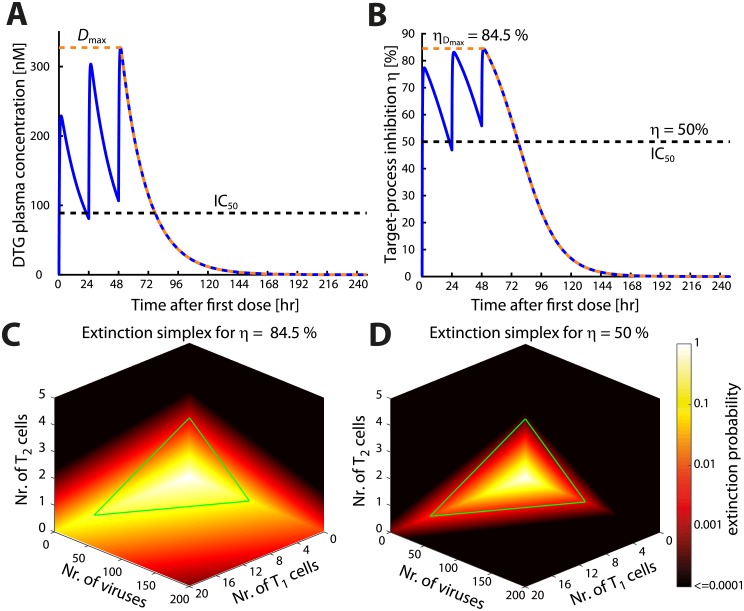
Adaptation of extinction simplex for pharmacokinetics. **A**: Exemplary DTG pharmacokinetics for 3days of 2mg oral DTG once daily. The blue line represents DTG plasma concentrations. The dashed orange line represents the function *D*_*max*_(*t*), which for a particular *t* returns the maximum DTG concentration achieved in any future time i.e *D*_*max*_(*t*) = max(*D*(*u*)) where *u* ∈ [*t*, ∞). The black horizontal dashed line marks the IC_50_ for DTG [38]. **B**: Instantaneous target-process inhibition (blue line) corresponding to the concentration-time profile in **A**. The orange line is the target-process efficacy profile for *D*_*max*_(*t*). The black horizontal dashed line marks *η* = 50%. **C**: Extinction simplex (viral infection state where the probability of viral extinction is greater than *ε*) corresponding to $\eta_{D_{max}}=84.5\%$. **D**: The extinction simplex corresponding to *η* = 50%. Panels **C**&**D** show the state space with three dimensions corresponding to number of free viruses, early-infected T cells (T_1_) and late-stage infected T cells (T_2_). The color varies from bright yellow denoting certain extinction, to black denoting an extinction probability less than 0.0001. The region enclosed by green lines is the extinction simplex in absence of antivirals.

**Fig 2:**
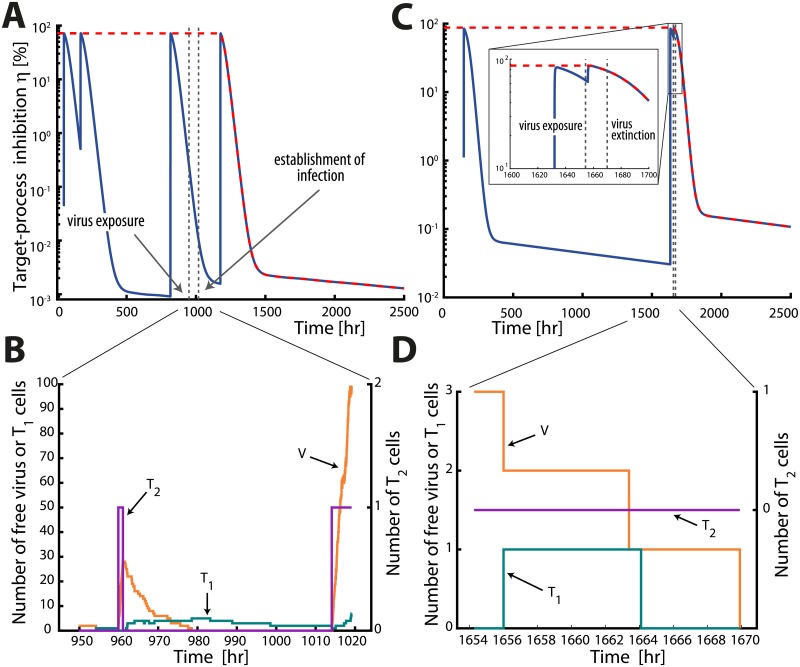
Examplary trajectories for time-varying drug effects. The left panels show an example of an infection event, whereas the right panels show an example of viral extinction for chronic PrEP with 2mg DTG and 5% adherence. Panels **A** and **C** depict the instantaneous target-process inhibition profiles and panels **B** and **D** depict the corresponding viral trajectories using the adapted EXTRANDE algorithm. Viral exposure occurs randomly during a 3 month period and is sampled from the distribution parameterized in [11] (Figure 2 therein). **A&C:** The blue lines depict the instantaneous target-process inhibition profiles *η*_*D*_(*t*). The dashed red line denotes the maximum target-process inhibition $\eta_{D_{max}}(t)$. The leftmost grey vertical dashed lines mark the time of viral exposure, whereas the rightmost lines marks the time point of either establishment of infection (panel **A**) or virus extinction (panel **C**). **B&D:** Stochastic trajectories of viral compartments (orange: free viruses, green: early-infected cells T_1_, purple: late-infected T_2_ cells) for the time after virus exposure and before virus infection/extinction. Stochastic simulations are stopped in panel **B** when the trajectories leave the extinction simplex and because of virus extinction in panel **D**.

The *extinction simplex* (eq (18)) then divides the entire state space of $Y\in {\mathbb{N}}^{3}$ into two sets: one where the extinction is possible (the probability of extinction exceeds *ε*) and one where irreversible infection occurred. Consequently, we can stop simulating and classify a trajectory as an ‘infection event’ whenever the trajectory leaves the *extinction simplex*. Moreover, the *extinction simplex* is dynamically adapted through the simulations by adaptation of *D*_max_(*t*). The effects of changing drug concentrations on the *extinction simplex* are illustrated in Fig 1 for a short course PrEP (‘PrEP on demand’) with dolutegravir in a virtual patient. The green triangle highlights the *extinction simplex* without drugs. Obviously, the *extinction simplex* in the absence of drugs is enclosed by the *extinction simplices* in presence of antivirals (i.e. viral extinction may still be possible in the presence of drugs when the viral population is substantial as in post-exposure prophylaxis PEP). Fig 2 shows two exemplary trajectories for the coupled pharmacokinetic-viral dynamic system, in case of low (5%) adherence to a 2mg oral dolutegravir regimen. Panels A & B show the instantaneous drug efficacy *η* (panel A) and the corresponding viral trajectory which is classified as an ‘infection event’ (panel B), whereas panels C & D show an exemplary trajectory where virus elimination occurs.

#### Infection of long-lived cells

It has been proposed that long lived- and latently infected cells denote a major barrier to the elimination of HIV and that they may be established early in infection [48–50]. Thus, if any of these compartments become infected after viral exposure, infection may be considered irreversible. During simulations we considered two parameters, $p_{M|a_{4}}=1.25\cdot 10^{-4}$ and $p_{L|a_{5}}=8\cdot 10^{-6}$ to assess whether a long-lived cell (e.g. macrophage) had been infected or whether a latently infected cell emerged. These parameter choices accurately reproduce viral decay kinetics during antiretroviral combination therapy, as shown in [24, 25] and recapture estimated reservoir sizes during chronic infection [2, 48]. I.e., during simulations, whenever reaction R4, or R5 fires, it is assessed whether a long lived- or latently infected cell emerged.

The complete pseudo-code of the adapted EXTRANDE algorithm is presented S1 Text.

### Simulation of pre- and post-exposure prophylaxis

Codes were written in MatLab R2016b (MathWorks, Natick, MA; v. 9.1, including optimization, parallel computing and statistics toolboxes). Individual pharmacokinetic model parameters for healthy individuals were drawn from the distributions defined by the parameter estimates from the final dolutegravir population pharmacokinetic model (Table 2; NONMEM $SIMULATION, n = 1000 individuals; eqs (12)–(14)). We then simulated individual pharmacokinetic profiles for the prophylactic schedule *S*_*D*_ under consideration using ode15s in MatLab. To simulate different adherence levels, a sequence of uniformly distributed random numbers with $r_{i}\sim U(0,1)$ was drawn and the *i*th dose was missed if *r*_*i*_ > adherence level. The number of viruses to be inoculated were drawn from a previously parameterized distribution that accurately resembles the relation between transmitter virus loads and recipient infection probabilities [11]. In brief, we used a two-stage process: First, we sampled the viral load VL in a potential transmitter. Earlier work [11] (Supplementary Note 4 therein) showed that the virus load distribution in potential transmitter populations [51] follows a log-normal distribution, i.e. VL~logN(μ,σ) with *μ* = 4.51 and *σ* = 0.98. Secondly, we used the virus load in the transmitter to determine the number of viruses *V*_0_ entering a replication-competent compartment in the virus-exposed individual using a binomial model: $V_{0}\sim B(f(\text{VL}),r_{\text{homo}})$ with *r*_homo_ = 3.71 × 10^−3^ for homosexual exposure [11], *f*(VL) = ||VL^*c*^||, where ||⋅|| is the next integer function, and *c* = 0.389 [52, 53]. For PrEP simulations with different adherence levels, a time of virus exposure was randomly drawn within a 3 month interval. The corresponding drug concentrations at this time and the number of transmitted viruses reaching a target cell compartment were used as the initial states for EXTRANDE and simulated until stopping criteria were satisfied, illustrated in Fig 2 for virus infection (panels A & B) and -elimination (panels C & D). For ‘PrEP on demand’ simulations, the time of virus exposure was fixed as indicated in the corresponding graphics. In case of PEP, virus was inoculated as stated above and the stochastic viral dynamics were simulated in the absence of drugs until the time of PEP initiation to determine the initial condition of the system and henceforth simulated until a stopping criterium was reached.

**Table 2:** Pharmacokinetic parameter estimates. The table displays the estimated pharmacokinetic parameter estimates for healthy individuals. Interindiviual variability (random effects) was included on drug clearance CL/*F*_bio_ and the volume of distribution *V*_*c*_/*F*_bio_. These parameters were log-normal distributed as outlined in the *Methods* section, eq (11), with coefficient of variation [%] $CV=100\cdot \sqrt{e^{\sigma^{2}}-1}$, where *σ*^2^ is the variance of the associated normal distribution. A covariance of $11.3\%=100\cdot \sqrt{e^{\sigma_{x,y}^{2}}-1}$ between *x* = CL/*F*_bio_ and *y* = *V*_*c*_/*F*_bio_ was estimated. The absorption rate constant was fixed [88] to 2.24h^−1^. Residual variability (eq (10)) was described by a combined proportional-additive model for healthy volunteers [*σ* = 0.213 (37.2%) and $\tilde{\sigma }=$ 0.0019 mg/L (40.9%), respectively] and a proportional error model for HIV-infected patients [*σ* = 0.402 (24.2%)].

parameter	value	unit	*CV* [%]
*V*_*p*_/*F*_bio_	0.73	L	-
*Q*/*F*_bio_	0.0082	L/h	-
CL/*F*_bio_	0.85	L/h	16.9
*V*_*c*_/*F*_bio_	17.7	L	16.4

In total, for each prophylactic strategy, 5000 simulations were run.

## Results

### Pharmacokinetics of oral dolutegravir

**Fig 3:**
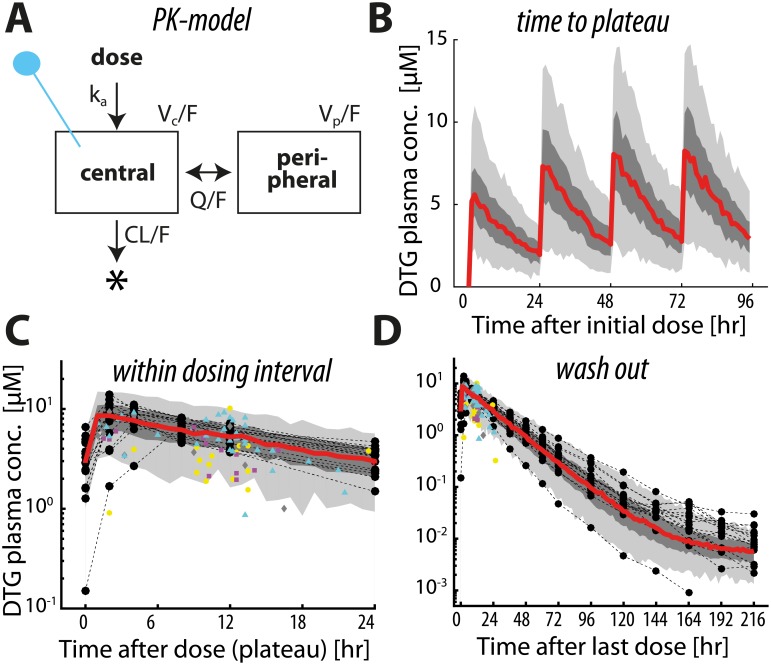
Population pharmacokinetics of dolutegravir (DTG). **A:** Pharmacokinetic model. Concentrations within the central compartment with bioavailability-adjusted volume *V*_*c*_/*F* correspond to measured plasma concentrations of DTG (indicated by the blue pin). Parameters *k*_*a*_, *Q*/*F*_bio_ and CL/*F*_bio_ denote the uptake and bioavailability-adjusted inter-compartimental and drug clearance rate respectively and *V*_*p*_/*F*_bio_ denotes the bioavailability adjusted volume of the peripheral compartment (which summarizes all ‘deep’ compartments, which are not in rapid exchange with the plasma). **B:** Predicted plasma concentration time profiles of dolutegravir (DTG) for the first four days after initiation of a once daily 50mg oral regimen (*N* = 300 virtual patients). The red line depicts the median predicted concentrations, whereas the dark- and light grey areas present the quartile range and 5–95% range respectively. Predicted (red line, grey areas) and measured plasma concentrations during 24h after drug intake in steady state (panel **C**) and after cessation of drug intake (panel **D**). Black circles and thin dashed lines represent DTG plasma concentration profiles in healthy volunteers (n = 17 concentration time profiles, 270 data points in total), whereas yellow circles, purple squares, grey diamonds and cyan triangles are DTG plasma concentration measurements in HIV patients (n = 39) observed 1, 2, 3 and 4 weeks after switching from efavirenz-based therapy to dolutegravir. Altogether, 354 plasma concentration measurements from 56 individuals are depicted.

A total of 354 plasma concentration measurements from 56 individuals were used to build the population pharmacokinetic (PK) model for dolutegravir (DTG). Healthy volunteers (N = 17) contributed rich PK profiles with a total of 270 samples taken between 0 hours (pre-dose) and 216 hours after a final DTG dose. In addition, eighty-four measurements, randomly drawn between 1-25.75 hours post-dose were available from HIV patients week 1, 2, 3 and 4 weeks post-efavirenz switch. A two-compartment pharmacokinetic model best described the data (Fig 1A) and was fitted in a Bayesian context to fully capture inter-individual pharmacokinetic variability. Following multivariate analysis, allometric scaling (centered on 70kg) of weight was considered as a fixed effect in the model. Different values of apparent oral clearance (CL/*F*_bio_) were estimated for DTG alone in healthy volunteers and in patients following 1, 2, 3 and 4 weeks post-treatment switch. Residual variability was described by a combined proportional-additive model for healthy volunteers and a proportional error model for HIV-infected patients. All parameter estimates for healthy volunteers are displayed in Table 2. The model was used to generate PK parameters of virtual patients populations, whose PK-profiles are summarized in Fig 3B–3D alongside observed DTG concentrations, Fig 3C and 3D.

As can be seen in Fig 3B, dolutegravir is rapidly absorbed after oral administration and maximal concentrations are achieved after *t*_max_ = 1.58 hours (population 5–95% range 1.53–1.63). Pharmacokinetics reach a steady state after about 4 doses. During steady state, minimum- (pre-dose) and maximum concentrations were *C*_min_ = 2918nM (1916–4336) and *C*_max_ = 8471nM (6353–11331) for 50mg oral DTG and the half life of the drug was 14.5h (5–95% range 13.5–15.9).

### Prophylactic utility of oral dolutegravir

**Fig 4:**
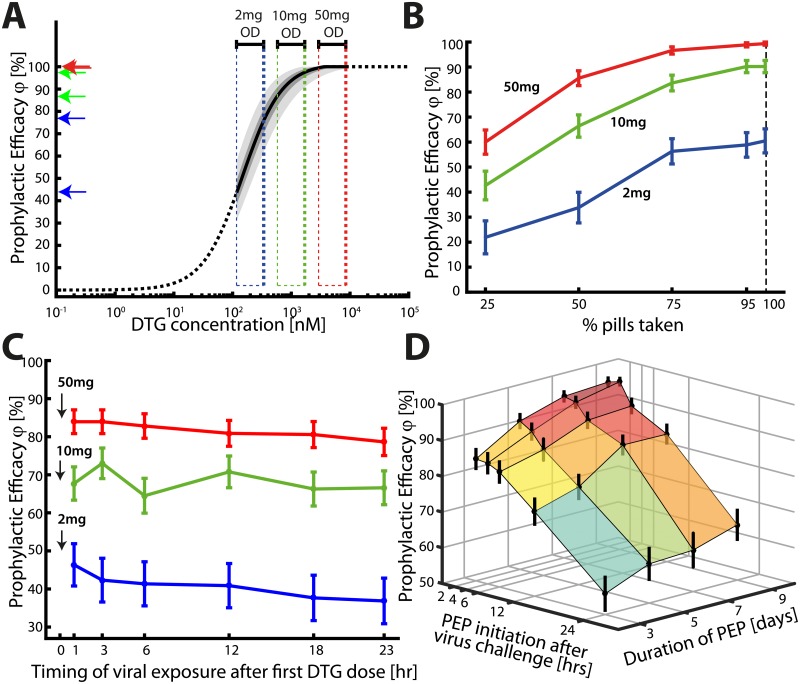
Efficacy of different DTG prophylactic regimen. **A:** Prophylactic utility of chronically administered oral DTG regimen (homosexual contact [11]). The red-, green and blue dashed boxes mark the considered concentration ranges of DTG [*C*_min_ (pre-dose), *C*_max_] achieved with 50, 10 and 2mg once daily (OD) oral dosing. The left pointing arrows at the y-axis mark the respective prophylactic efficacy ranges. **B:** Prophylactic efficacy of chronically adminstered oral DTG regimen with varying adherence levels. The red-, green- and blue lines denote mean prophylactic efficacy for a 50mg, 10mg and 2mg oral DTG regimen. Errorbars depict the 95% confidence bounds for the ensemble estimate, computed using Greenwoods formula. **C:** Prophylactic efficacy of DTG for ‘PrEP on demand’. Only three doses of oral DTG were ingested at 0, 24 and 48 hours. Homosexual viral exposure occurred within the first dosing interval at either 1, 3, 6, 12, 18 or 23 hours after initiating ‘PrEP on demand’. The red, green and blue lines represent the mean prophylactic efficacy for ‘PrEP on demand’ using 50-, 10 or 2mg respectively, where error bars denote the 95% confidence bounds for the ensemble estimate, computed using Greenwoods formula. **D:** Prophylactic efficacy for ‘post exposure prophylaxis’ (PEP) with 50mg DTG for various durations of PEP (y-axis; 3, 5, 7 and 9 days) and delayed initiation of PEP after homosexual viral exposure (x-axis; 2, 4, 6, 12 and 24 hours). Error bars mark the 95% confidence bounds computed using Greenwoods formula.

Fig 4A shows the relation between the plasma concentration of DTG and its prophylactic efficacy after homosexual virus exposure. For these simulations, the number of viruses reaching a replication-competent compartment after homosexual contact were sampled from a previously parameterized virus exposure model [11] (Fig.2 and Supplementary Note 4 therein). The estimated concentration ranges achieved at steady state for 2-, 10- and 50mg oral DTG once daily were 117–339, 583–1694 and 2918–8471nM respectively as indicated on the y-axis of Fig 4A. Within this concentration range, the median prophylactic efficacy for once daily 2mg ranged from 43.6 to 75.7%. For 10mg, efficacies ranged from 87.1 to 97.5%, and for 50mg almost complete (99.5 to 100%) protection was achieved. The estimated concentrations to prevent 50- and 90% infections, $EC_{50}(\hat{V})$ and $EC_{90}(\hat{V})$, were 145.18 and 722.23nM respectively.

### Sensitivity to incomplete medication adherence

During pre-exposure prophylaxis, medication adherence may be incomplete. Fig 4B displays the prophylactic efficacy of once daily 50mg, 10mg and 2mg oral dolutegravir, considering varying levels of adherence (25-, 50-, 75-, 95- and 100% of doses taken). Viral challenges were simulated to randomly take place during a 3 month interval with inoculum sizes drawn from a previously parameterized distribution [11], see Fig 2 for two examples. The mean predicted prophylactic efficacies for 50mg with 25-, 50-, 75-, 95- and 100% adherence were 60% (95% confidence bounds: 55.15–64.84), 85.54% (82.58–88.50), 96.63% (95.19–98.07), 98.88% (98.04–99.71) and 99.36% (98.73–99.99), respectively. Notably, the prophylactic efficacy of 50mg oral DTG becomes saturated, and exceeds 95%, if at least 75% of the pills were taken. Conversely, 2mg and 10mg oral dolutegravir allow for considerable residual infection events and 2mg oral dolutegravir efficacy increases almost linear with increasing adherence levels.

### PrEP on demand/event-based dosing

‘PrEP on demand’ denotes a short-term pre-exposure prophylaxis, initiated only a few hours before a potential viral exposure. This strategy has recently been evaluated in the IPERGAY study using Truvada [54]. We simulated a dosing scheme similar to the IPERGAY protocol [54]: An individual at risk initiates PrEP only a few hours before a potential viral exposure and takes two consecutive doses 24 and 48hours after the first dose. Fig 4C depicts the predicted prophylactic efficacy of DTG when taken ‘on demand’. Population pharmacokinetic profiles for ‘PrEP on demand’ using 50mg are depicted in Fig 3A. The mean prophylactic efficacies for 50mg varied, depending on the timing of the first dose with respect to viral exposure, between 78.63–83.93% (95% confidence bounds: 75.05–87.05), for 10mg between 64.49–73.01% (59.92–77.02) and for 2mg it was 36.86–46.34% (30.87–51.90). The prophylactic efficacy decreased with decreasing dose and also with increasing time difference between the initiation of ‘PrEP on demand’ and viral exposure, clearly visible for 50 and 10mg. This trend is opposite to the trend for ‘PrEP on demand’ with Truvada [11]. A reason for this is the fast uptake of systemic DTG (compare Fig 4B), whereas the Truvada’s active components tenofovir diphosphate (TFV-DP) and emtricitabine triphosphate (FTC-TP) require intracellular phosphorylation after cellular uptake of the parent compound, which delays the time until maximal concentrations are achieved at the target site [31, 55]. Since DTG does not need to undergo any chemical modification, and according to ‘free drug hypothesis’ the unbound intracellular concentration largely reflects the unbound plasma concentration, implying that the drug rapidly reaches the target site.

### Post-exposure prophylaxis (PEP)

Lastly, we wanted to assess the efficacy of 50mg oral DTG in preventing infection when taken as post-exposure prophylaxis (PEP). We assessed the prophylactic efficacy with regard to different durations of PEP and with regard to the timing of initiation after virus exposure in Fig 4D. Fig 4D indicates that 50mg oral DTG can effectively prevent infection (> 80%) when initiated shortly (within 6 hours) after exposure and when continued for as long as possible. The graphics indicate that the efficacy starts to drop when PEP is initiated later than 6 hours and when it is taken shorter than 7 days. Also, our simulations suggest that initiating the prophylaxis earlier has a more pronounced effect than prolonging PEP, arguing for the immediate start of PEP in case of known- or suspected HIV exposure.

### Comparison with Truvada

We previously estimated that once daily PrEP with Truvada provides ≈ 96% protection in fully adherent individuals [11]. While it is difficult to quantify PrEP adherence clinically [56], a surrogate measure is often calculated based on the percentage of individuals with detectable drug. Corresponding clinical efficacy estimates in apparently highly adherent individuals were 86-100% in the IPERGAY study [57], 58-96% in the PROUD study [6] and 96% in the Partners PrEP OLE study. In comparison, we predicted almost complete (99-100%) protection when 50mg DTG was taken once daily for prophylaxis. The VOICE [58] and FEM-PrEP [59] studies indicated that Truvada may not prevent infection in poorly adherent individuals, i.e. if ≈ 30% of individuals had detectable drug. In comparison, with 25% adherence to once daily 50mg DTG, we estimated about 60% protection and over 85% protection if at least half the drugs were taken. For ‘PrEP on demand’ with Truvada, we recently estimated [11] that about 74–92% infections can be averted, depending on the time of viral exposure relative to the initiation of Truvada dosing. The corresponding efficacy estimate in the IPERGAY trial was 86% [54], in line with our previous work. Herein, we predicted that ‘on demand’ PrEP with 50mg DTG is non-inferior to Truvada, providing 78.63–83.93% protection. Lastly, while PEP with Truvada is not recommended due to the slow intracellular accumulation of pharmacologically active NRTI-triphosphates, PEP with 50mg DTG can prevent about 80% infections when initiated no later than 6hours post exposure. In summary, our simulations indicate that prophylaxis with 50mg DTG is non-inferior to Truvada and that it may outperform Truvada when individuals’ medication adherence substantially deviates from a once daily PrEP protocol, as in the case of poor adherence and post-exposure prophylaxis.

## Discussion

While pre-exposure prophylaxis with Truvada can prevent sexual HIV-1 transmission, it has severe limitations that are to be overcome by next-generation PrEP regimen [8]. However, drug candidate selection for next-generation PrEP and selection of administration schemes in clinical trials are prone to high failure rates. One reason is the poor translatability of animal- and ex vivo/in vitro experiments, whereas the statistical requirement of large sample sizes (N > 1000 individuals) and long trial durations to detect prophylactic efficacy *clinically* leads to exorbitant costs. Thus, there is an unmet need for tools to *a priori* assess the utility-, select and prioritise next generation PrEP candidates for human trials.

Our intent was to develop a method that integrates pharmacokinetic and pharmacodynamic (PK/PD), as well as viral characteristics (inoculum size, timing of exposure) to *a priori* assess prophylactic strategies against HIV. Such integrative framework allows for the intelligent design of once-daily, episodic PrEP, as well as simulating the on- and offset of event-driven PrEP [54] or long-acting injectable PrEP formulations [60–62]. Moreover, it allows to assess how stable a prophylaxis is when individuals poorly adhere to the planned prophylaxis scheme, i.e. when individuals miss dosing events or start to take the drugs shortly before- or only after exposure to HIV. The use of population pharmacokinetic models allows for an accurate description of the observed pharmacokinetic variability within- and across patients, which can be used to assess the effectiveness of PrEP coverage in metabolically diverse population (Figs 3 and 4). While the attributes which make any compound favourable *in the clinic* extend beyond PK/PD, our approach is particularly useful to rule out- or prioritize PrEP candidates and/or strategies for further *clinical* investigation.

To enable this ambitious goal we had to develop the theoretical basis that would enable us to accurately predict infection/extinction events for any arbitrary prophylaxis dosing regimen. The theoretical context is to compute solutions of the chemical master equation (CME), which is intractable due to the *curse of dimension*. Most naturally, instead of directly solving the CME, it is possible to sample trajectories using Monte Carlo techniques such as the stochastic simulation algorithm (SSA) [47] and to empirically reconstruct $\mathbb{P}(X_{t}=x_{i})$ from the trajectories. However, it is not clear when to classify a trajectory as an infection event. Moreover, stochastic simulations become inefficient when reaction propensities are high, which is typically encountered in a large copy number regimen, e.g. when *X*_*t*_ >> 1. To solve the latter issue (large copy number regimen), a number of hybrid methods have been proposed that partition a system *X* = (*Y*, *Z*) into species *Y* that are represented by a discrete-stochastic (CME) model and species *Z* that are approximated by their concentrations [63, 64]. Since *Z* then evolves on an infinitesimally small time scale, a natural consequence of this partitioning is that stochastic propensity functions *a*(*Y*_*t*_, *Z*_*t*_) evolve between two *stochastic* reaction firings (*t*, *t* + *τ*). Thus, numerically exact computation requires to solve an integration problem to compute *a*(*Y*_*t*_, *Z*_*t*+*u*_). In sampling-based methods, i.e. the integral-based methods employed in [25, 55], frequent initialization of numerical integrators can become a major computational burden (i.e. *τ* small). Direct hybrid methods [65–67] overcome this problem, however these methods still require to directly solve the CME part, which can be prohibitively large (involving thousands of states), limiting their applicability. In S2 Fig we show a comparison of simulations results obtained by integral-based (e.g. as used in [25, 55]) sampling methods vs. the EXTRANDE method, as well as their respective run times. As expected, the results are identical, while the simulation time is lower for EXTRANDE.

The method we are using is the recently developed EXTRANDE (extra reaction algorithm for networks in dynamic environment) method [12]. The EXTRANDE approach is based on a rejection, or thinning and allows for the numerically exact simulation of intrinsically stochastic kinetics embedded in a dynamical environment, i.e. the stochastic sub-system *Y* may constitute time-dependent propensities that are affected by the dynamic environment *Z*. We adapted EXTRANDE to allow the numerically exact prediction of infection/virus clearance in the context of time-varying drug inhibition. The key adaptation was to define proper stopping criteria that classify trajectories as infection events. To this end, utilizing results from a related article [23], we were able to compute and dynamically update an *extinction simplex* (eq (18)), i.e. a part of the state space in which extinction can occur, see Fig 1. Whenever a trajectory leaves the *extinction simplex*, it is safe to classify it as an infection event and to stop the simulation.

Classifying trajectories as infection events is not a straightforward choice: Using arbitrary thresholds *Y*_*tr*_ = [*V*_*t*_ = *c*_0_, *T*_1,*t*_ = *c*_1_, *T*_2,*t*_ = *c*_2_]^*T*^ can significantly distort predictions (e.g. ‘PrEP efficacy’) or increase computational run-time: While too small thresholds overestimate the number of infection events, large thresholds increase the computational run-time unnecessarily, since stochastic simulations become very inefficient when *Y* >> 1. Furthermore, there is no control- or knowledge of the numerical error made (the probability to falsely classify a trajectory as an infection event). Using the *extinction simplex* method proposed herein, it is guaranteed that the probability to falsely classify a trajectory stays below a user-defined threshold *ε* << 1. Moreover, the algorithmic run-time is optimal for providing this user-defined precision.

Particularly during ‘PrEP on demand’ or ‘PEP’ simulations, dynamic adaptation of the *extinction simplex* can be algorithmically harnessed: Note that there is a positive relation between the size of the *extinction simplex* and the drug concentration *D* (compare Fig 1). After the last dosing events in ‘PrEP on demand’ or ‘PEP’ simulations the size of the extinction simplex shrinks as the drug concentration tapers, making it more likely that stopping criteria are met, which minimizes runtime.

We used our framework to assess the utility of dolutegravir (DTG), which may be suitable for prophylaxis, since it has a good safety profile [68], a high resistance barrier [69] and a long half life in the blood plasma. Foremost, utilizing *in vitro* and *in vivo* parameters in our mathematical framework, we estimated that concentrations of $EC_{50}(\hat{V})=145.18$ and $EC_{90}(\hat{V})=722.23\text{nM}$ prevent 50- and 90% infections respectively. These concentrations can guide dosing and release kinetics of nanoformulated long-acting dolutegravir, which is currently in preclinical development [62]. Moreover, as soon as human pharmacokinetic data is available, our framework can easily be adapted to predict the PrEP utility of the long-acting formulation (by updating the pharmacokinetic model). As an example, we focussed on *oral* DTG herein, for which we had sufficient pharmacokinetic data to build a population PK model. Combining this model with the EXTRANDE framework, allowed to assess different prophylactic strategies: Overall, our simulations suggested that oral 50mg OD DTG may have a potential for PrEP with an estimated efficacy of 99 to 100% (perfect adherence). For comparison, we previously estimated that Truvada may prevent 96% of infections when taken once daily (perfect adherence) [11]. Our model suggests that DTG’s protective efficacy remains high (> 80%) even at adherence levels as low as 50%. This apparent forgiveness to poor adherence is due to DTG’s prophylactic potency rather than its halflife: I.e. if most DTG doses are taken, concentrations ranges are achieved where the concentration-prophylaxis profile is saturating (compare Fig 4A). Consequently, concentration changes do not proportionally translate into changes in prophylactic efficacy. In event-driven PrEP, we predicted that prophylactic efficacy reflects the drug profile in the blood plasma (compare Fig 3), which is characterized by a rapid absorption (*t*_max_ ≈ 1.58 [h]) and slow elimination. This is in contrast to tenofovir-emtricitabine (Truvada) whose activity is not reflected by their plasma levels, since these drugs require conversion to intracellular diphosphates [31, 55] to exert their antiviral activity, delaying the overall onset of activity. The main advantage of DTG over Truvada is in the context of post-exposure prophylaxis (PEP), where we predicted that it can potently prevent infection if initiated no later than 6hours post-exposure and taken for at least 5 days. We do not suggest to use single drug PEP with DTG; the sole purpose was to determine whether DTG would be effective, if individuals fail to take it *before* virus exposure. This kind of assessment allows to quantify the risks of prophylaxis in situations where the regimen is not taken as intended and moreover provides a scientific basis to include it in PEP multi-drug combinations.

Our model has several limitations, but also a number of important advantages. Our simulations do not take into account drug concentrations at the site of mucosal exposure (e.g. cervix, rectum) [60, 70]. These concentrations have, however, not been validated as targets for successful prevention or treatment, whereas data exist (albeit limited) for plasma drug concentrations. Instead, we modelled based on *unbound* concentrations, in line with the widely accepted ‘free drug hypothesis’, stating that unbound concentrations at the target site are responsible for pharmacological action. For drugs highly bound to plasma protein (> 90%), naturally since plasma protein concentrations are lower in tissues other than plasma, their *total* concentrations at sites other than the plasma are magnitudes lower [70]. Strikingly, however, the unbound plasma concentrations coincide with the unbound tissue concentrations [71], strongly arguing for the validity of the ‘free drug hypothesis’ [40, 41]. Therefore, throughout the work, we assumed, according to the ‘free drug hypothesis’ [39] that the unbound concentrations in plasma and at the target site coincide. Note that dolutegravir is highly lipophilic (logP ≈ 2), enabling the *unbound* drug to rapidly cross cellular membranes, generating an equilibrium between the *unbound* drug on either side of the cellular membrane [42].

We estimated the probability of virus clearance (and the prophylactic efficacy *φ*) as a function of the number of viruses ultimately reaching a target cell environment after sexual exposure, and not as a function of mucosal exposure. The utilized virus exposure model [11] is calibrated to reflect the per-contact infection risks for typical transmitter virus loads and different routes of sexual exposure exposure. However, it should be noted that in an accompanying article [23], we also observed that increasing the inoculum size decreases the prophylactic efficacy, i.e. estimates of prophylactic efficacy depend on the route of transmission: For example, if exposure to HIV occurs via blood transfusion (large inoculum size), most prophylactic drugs may fail to offer protection.

Our framework can be adapted or developed in a number of ways. The separate impact of treatment as prevention [72] (reduction of donor virus load to decrease contagiousness) versus prophylactic efficacy in the recipient individual can be simulated by calibrating the virus inoculum distribution [11]. The effect of PrEP on *resistance transmission* from a donor to a recipient can be incorporated in the framework by increasing IC_50_ in eq (9) (fold resistance) and possibly reducing certain reaction constants (fitness deficits). Likewise, the effects of PrEP on *resistance emergence* can be considered. However, during the *early* events after virus exposure (when infection can still be averted), the population size may be too small for resistance to appear *de novo* in the exposed individual. For example, a particular point mutation appears with probability 1 − (1 − *μ*)^*n*^, where *μ* ≈ 2.2 ⋅ 10^−5^ [73] is the per base mutation rate of HIV per cell infection (= reverse transcription event) and *n* the number of cell infection events. According to these numbers, it requires ≈ 30000 cells to be infected for resistance to arise with 50% probability. Considering these numbers, a likely scenario for *de novo* resistance to appear is when PrEP had not been taken at the time of exposure, such that the infection expanded exponentially and a resistant mutant may have been generated at random. When PrEP is (re-)initiated at some later time it could provide the necessary pressure to select out the resistant type from the quasispecies population. Modelling these events is out of the scope of the recent work, as it requires distinct (and more coarse) simulation approaches, e.g. [25, 74].

It is well known that the establishment of a latent reservoir is the major barrier to viral extinction during treatment [49] and this reservoir may be established as early as 3 days post infection [48, 50]. We considered infection of long lived cells during our simulations, as outlined in the *Methods* section. I.e., whenever long-lived cells became infected during simulations, viral extinction was considered infeasible. Notably, there are two sources: one with a half life of ≈ 14 days is responsible for the second phase of viral decay that can be observed in infected patients on combination treatment [75, 76]. This reservoir has been attributed to infected macrophages [75, 77]. Based on the mentioned half life of this reservoir, it would require $T(x)=-\frac{\text{ln}(1-x)}{\text{ln}(2)/t_{1/2}}$ days to eliminate a single cell with probability *x* under complete virus inhibition, e.g. ≈ 45 days to eliminate a single infected macrophage with 90% probability (*x* = 0.9) and over 90 days to eliminate this reservoir with 99% probability. Latent infected T cells decline even slower, with a half life of ≈ 6-44 month [78–80]. This reservoir is partly responsible for the third phase of ‘decay’ and is assumed to prevent HIV cure during effective combination treatment.

In summary, we have developed an innovative modelling approach to *a priori* assess prophylactic roll-out strategies by fully integrating individual PK/PD profiles and viral dynamics into a hybrid stochastic-deterministic framework. We used this framework to assess the prophylactic efficacy of the second-generation integrase inhibitor dolutegravir with respect to poor adherence, in event-driven prophylaxis and post-exposure prophylaxis. Overall our simulations showed that oral prophylaxis with 50mg DTG is non-inferior to Truvada and has profound advantages with respect to post-exposure prophylaxis. Moreover, we predicted that concentrations above EC_90_ = 722.23*nM* can prevent > 90% infections after sexual exposure. These target concentrations can guide loading doses for novel long-acting nanoformulations of DTG [62]. By adapting the pharmacokinetics model, the framework can easily be used to predict the prophylactic utility of other candidate drugs currently under development, such as oral maraviroc (MVC) [81], and raltegravir (RAL) long-acting injectable rilpivirine [60] or cabotegravir [61], or it may be adapted to predict vaccine efficacy.

## Supporting information

S1 FigSchematic of the HIV replication cycle and mechanism of interference by dolutegravir.The viral dynamics model (adapted from [24, 25]) consists of free infectious viruses *V*, early infected T-cells (T_1_), productively infected T-cells (T_2_) and uninfected T-cells *T*_*u*_ = *const*.. Early infected T-cells (T_1_) and productively infected T-cells (T_2_) denote T-cells prior- and after proviral integration respectively, where the latter produces virus progeny. Free viruses are cleared by the immune system with a rate constant CL. Further, free viruses can be also cleared during unsuccessful T-cell infection CL_*T*_ through the destruction of essential viral components of the reverse transcription-, or pre-integration complex [34, 35]. The term *β* represents the lumped rate of infection of T-cells, including the processes of virus attachment to the cell, fusion and reverse transcription, leading to an early infected cell T_1_, before proviral integration. The term *k* denotes the rate by which early infected T_1_ cells are transformed into productively infected T_2_ cells, involving proviral integration and cellular reprogramming. The term N_T_ denotes the rate of production of infectious virus progeny by productively infected T_2_ cells. The terms $\delta_{T_{1}}<\delta_{T_{2}}$ denote the rates of clearance of T_1_ and T_2_ cells respectively and *δ*_PIC_ denotes the rate of intracellular destruction of the pre-integration complex. Parameters are summarized in Table 1. The second-generation integrase inhibitor dolutegravir (DTG) prevents proviral integration and consequently decreases *k* by a factor (1 − *η*_*D*_). Long-lived and latently infected cells are implicitly considered in the model, i.e. the parameters $p_{M|a_{4}}$ and $p_{L|a_{5}}$ denote the conditional probabilities that a long lived- or a latent cell, which are a barrier to viral eradication, become infected. These rare events define algorithmic stopping criteria (irreversible infection) when modelling complex prophylactic regimen, e.g. long-term pre-exposure prophylaxis with inadequate adherence. DTG: dolutegravir.(EPS)

S2 Fig Comparison of EXTRANDE (green) and integral-based hybrid stochastic-deterministic simulation (red). **A:** Prophylactic efficacy for ‘post exposure prophylaxis’ (PEP) with 50mg DTG for various durations of PEP (x-axis; 3, 5, 7 and 9 days) and when initiated 24 hours after homosexual viral exposure. Error bars mark the 5-95% range computed using Greenwoods formula. **B:** Corresponding simulation run times on an intel i7 core with 2.5Ghz and 16 GB RAM. **C:** Simulation run times for the subset of simulations where infection occurred. Median (25-75 quartile ranges) runtime (sec) for 3 days PEP: 8.524 (2.4–20.5) vs. 10.548 (2.4–26.1); 5 days PEP: 10.0 (2.8–25.9) vs. 23.0 (6.8–48.1); 7 days PEP: 17.4 (5.3–35.4) vs. 25.5 (10.1–57.4) and 9 days PEP: 26.1 (7.6–57.3) vs. 34.1 (12.0–82.3).
(EPS)

S1 Text The supplementary text contains a complete pseudo-code for the adapted EXTRANDE algorithm used for simulating initial viral dynamics during prophylaxis. (PDF)
