# Supplementary material for: Hybrid stochastic framework predicts efficacy of prophylaxis against HIV: An example with different dolutegravir prophylaxis schemes
Source: PLoS Comput Biol. 2018 Jun 14;14(6):e1006155. doi: 10.1371/journal.pcbi.1006155 (PMC6001963; doi:10.1371/journal.pcbi.1006155)
Supplement: S1 Text — (PDF) [file pcbi.1006155.s003.pdf]

Supplementary Text S1:
Pseudo-code of the adapted EXTRANDE algorithm

Precomputation :
Compute target-site pharmacokinetic profile D_s for $s \in [0, T]$
Initialization :
$t = 0$ # Initial time of the system
$Y = Y_0$ # Initial state of the stochastic system
while *true* **do**
    # Determine upper bound for the sum of reaction propensities
    $B = a_0(Y_t, \varnothing) = \sum_{k=1}^{K} a_k(Y_t, \varnothing)$ # Sum of reaction propensities in absence of antivirals
    # Generate putative reaction time i.e $\tau \sim Exp(1/B)$:
    $r_1 \sim \mathcal{U}(0, 1)$ # Sample from a uniform distribution
    $\tau \leftarrow \frac{1}{B} \cdot \log\left(\frac{1}{r_1}\right)$ # Transformation to an exponential distribution
    $t \leftarrow t + \tau$ # Update time
    $a_0 \leftarrow \sum_{k=1}^{K} a_k(Y_t, D_t)$ # Sum of all stochastic reaction propensities at time t
    $r_2 \sim \mathcal{U}(0, 1)$ # Sample from a uniform distribution
    if $a_0 \geq B \cdot r_2$ **then**
        # 'Acceptance Step' - a reaction fires changing the state.
        # Choose the reaction, i.e.
        the smallest positive integer j such that $\sum_{k=1}^{j} a_k(Y_t, D_t) \geq B \cdot r_2$
        if *reaction R4 or R5 is chosen* **then**
            $r_3 \sim \mathcal{U}(0, 1)$
            if $r_3 \leq p_{M|a_4}$, *respectively* $r_3 \leq p_{L|a_5}$ **then**
                # a long lived/latently infected cell emerged.
                Stop the simulation
            end
        end
        $Y_t \leftarrow Y_t + \nu_j$ # Update the state of the system
        if $Y_t = \mathbf{0}$ **then**
            # Extinction event - the trajectory has reached the absorbing extinction state.
            Stop the simulation
        else
            # Compute the extinction probability of state Y_t and drug concentration D_{max}
            Compute D_{max} for the current time t
            if $P_E(Y_t, D_{max}) < \varepsilon$ **then**
                # Infection event
                Stop the simulation
            else
                # The trajectory at time t is within the extinction simplex.
            end
        end
    else
        # 'Rejection Step' - Extra reaction fires without changing the state.
    end
end

Algorithm 1: Adapted **Ext**ra **R**eaction **A**lgorithm for **N**etworks in **D**ynamic **E**nvironments (EXTRANDE) for estimating the infection/extinction probability for time-varying drug effects.
